# MicroRNA transcriptome profiles during swine skeletal muscle development

**DOI:** 10.1186/1471-2164-10-77

**Published:** 2009-02-10

**Authors:** Tara G McDaneld, Timothy PL Smith, Matthew E Doumit, Jeremy R Miles, Luiz L Coutinho, Tad S Sonstegard, Lakshmi K Matukumalli, Dan J Nonneman, Ralph T Wiedmann

**Affiliations:** 1 USDA/ARS Meat Animal Research Center, Clay Center, NE, USA; 2 Department of Animal Science, Michigan State University, East Lansing, MI, USA; 3 University of Sao Paulo-ESALQ, Piracicaba, SP, Brazil; 4 USDA/ARS Beltsville Area Research Center, Beltsville, MD, USA; 5 Bioinformatics and Computational Biology, George Mason University, Manassas, VA, USA

## Abstract

**Background:**

MicroRNA (miR) are a class of small RNAs that regulate gene expression by inhibiting translation of protein encoding transcripts. To evaluate the role of miR in skeletal muscle of swine, global microRNA abundance was measured at specific developmental stages including proliferating satellite cells, three stages of fetal growth, day-old neonate, and the adult.

**Results:**

Twelve potential novel miR were detected that did not match previously reported sequences. In addition, a number of miR previously reported to be expressed in mammalian muscle were detected, having a variety of abundance patterns through muscle development. Muscle-specific miR-206 was nearly absent in proliferating satellite cells in culture, but was the highest abundant miR at other time points evaluated. In addition, miR-1 was moderately abundant throughout developmental stages with highest abundance in the adult. In contrast, miR-133 was moderately abundant in adult muscle and either not detectable or lowly abundant throughout fetal and neonate development. Changes in abundance of ubiquitously expressed miR were also observed. MiR-432 abundance was highest at the earliest stage of fetal development tested (60 day-old fetus) and decreased throughout development to the adult. Conversely, miR-24 and miR-27 exhibited greatest abundance in proliferating satellite cells and the adult, while abundance of miR-368, miR-376, and miR-423-5p was greatest in the neonate.

**Conclusion:**

These data present a complete set of transcriptome profiles to evaluate miR abundance at specific stages of skeletal muscle growth in swine. Identification of these miR provides an initial group of miR that may play a vital role in muscle development and growth.

## Background

Functionally important small RNAs were first described in nematodes in 1993 [1,2]. However, it was not until 2001 that researchers began to understand the function of this family of RNAs that includes microRNA (miR) and to recognize that their significance was not confined to lower order organisms [3,4]. The small RNA classified as miR are short sequences, 18–26 nucleotide long, encoded by nuclear genes that produce characteristic stem-loop RNA structures when transcribed. During processing from the primary transcript, the mature miR sequence is loaded into an RNA:protein complex known as the "RNA induced silencing complex" (RISC) [5,6]. The sequence of the miR loaded in the complex targets the RISC to specific binding sites in the 3' untranslated region of mRNA transcripts, resulting in either degradation of the miR:mRNA complex or translocation to processing bodies. In either case, association of RISC with mRNA causes decreased translation of the targeted gene product [6,7]. As a result of decreased translation of their cognate targets, miR have been reported to guide developmental decisions including cell fate, cell cycle progression, apoptosis, adipocyte differentiation, and processes that alter muscle development and growth including myoblast proliferation, differentiation, and skeletal muscle hypertrophy [8-15].

The objective of the current research was to evaluate miR transcriptome profiles during skeletal muscle development in swine. MicroRNA were initially reported to have a role in skeletal muscle development utilizing mouse, drosophila, and zebrafish models. Three muscle-specific miRNA (miR-1, miR-133, and miR-206) were identified to increase in abundance during muscle cell differentiation [10,16,17]. However, these miRNA have been reported to regulate different stages of myogenesis [12,13,15]. MiR-133 increases proliferation of C_2_C_12 _myoblasts, whereas miR-206 and miR-1 promote differentiation [16]. Research in livestock models has begun to evaluate the role these miRNA have in skeletal muscle development. Expression of the muscle regulatory factor, myogenic factor 5, has been reported to regulate miR-1 and miR-206 transcription level in a chicken cell culture model [18]. In addition, over-expression of fibroblast growth factor-4 has been reported to decrease miR-206 abundance, resulting in developmental changes in the somite of developing chicken embryos [19]. Muscle-specific miR have also been reported to regulate a gene that directly impacts economic traits in livestock [20]. A mutation in the myostatin gene of heavily muscled Belgian Texel sheep creates a target site for miR-1 and miR-206 containing RISC complexes in the 3' untranslated portion of the transcript, resulting in decreased translation of the myostatin protein and consequent increase in muscle mass.

With the dramatic increase in identified miR sequences for multiple species including livestock species, a public database dedicated to the cataloguing of predicted and experimentally observed miRs has been developed (miRbase) [21-23]. In human, 678 miRs have been described (miRbase release 11.0, April 2008). However, genomic sequence scans and miR cloning results indicate that the actual total number of human miRs may be closer to 800 [24]. Comparative analysis of these sequences indicates that they are highly conserved among species as diverse as nematodes and mammals, supporting the hypothesis that they are of central importance to biological processes. In addition, expression of miR genes is tightly regulated spatially among tissues and temporally within tissues during development in all species studied, indicating the importance of determining miR transcriptome profiles to fully understand their biological importance [25-27]. In order to identify miR and determine their role in skeletal muscle of livestock, we evaluated miR transcriptome profiles at specific stages of muscle development including proliferating satellite cells, three stages of fetal growth, day-old neonate, and the adult.

## Results and Discussion

### Validation of miR cDNA libraries

The method of miR identification and quantification by cloning and sequencing has been utilized in numerous reports of miR biology [28]. It is particularly advantageous to use this method when working in species with poorly characterized genomes, since variation in miR sequences between species has become apparent [27,29,30].

**Figure 1:**
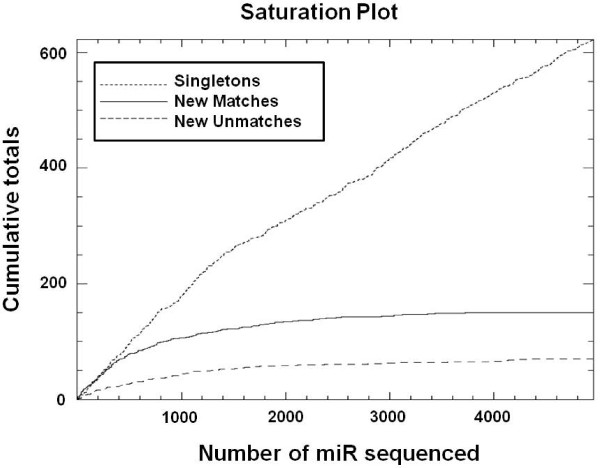
**Saturation plot of microRNA libraries**. Saturation plots were created from the neonatal muscle sample to determine saturation of the signal as an indicator that abundance levels would remain constant as new results were added. A total of 5,000 observed miR were evaluated. The supply of unique singletons is represented by the small dashed line, while the known and unknown miR are represented by the solid black line and large dashed line, respectively.

Our initial goal was to obtain complete transcriptome profiles of miR abundance in skeletal muscle, and to accurately measure changes in the level of abundance for these miR between developmental stages. Time points evaluated included proliferating satellite cells (4^th^, 5^th^, and 6^th ^passage), three stages of fetal development (60, 90, and 105 day-old fetus), day-old neonate, and adult. After identification of miR, accurate quantification of changes in miR abundance level between libraries and stages of skeletal muscle development was imperative. For all species, the dynamic range and precision obtained with enumerating each sequence observed enables a more quantitative description of miR abundance levels. Converting this precision to accuracy, requires running enough samples to saturate the signal until the abundance levels stay constant as new sequences are added. Initially, data from the neonatal muscle profiles were used to determine miR signal saturation as an indicator that abundance level of miR would remain constant as additional data were added (Figure 1). Although the number of unique singletons was far from exhausted after 5,000 observed putative miR, the number of new sequences observed for known and unknown miR began to plateau, suggesting that the singletons were either rare miR, represent contamination of the tissue with trace amounts of other tissues, or are sequence artifacts. Therefore, the data sets consisting of several thousand miR sequences were determined to be extensive enough to capture the diversity of miR abundance and estimate relative steady state levels in the developmental stages examined. Similar plots (not shown) were created from proliferating satellite cell profiles of the 4^th ^and 5^th ^passage. As with the neonate, abundance levels of known and unknown miR were well defined once a few thousand miR had been sequenced, as estimated abundance levels based on the first 3,000 sequenced clones from the 4^th ^and 5^th ^passage satellite cells agreed with estimates for the full library of 8,832 clones within 10%. Together, these results provide confidence that the number of clones sequenced provide an accurate representation of the miR transcriptome profiles of porcine skeletal muscle.

**Figure 2:**
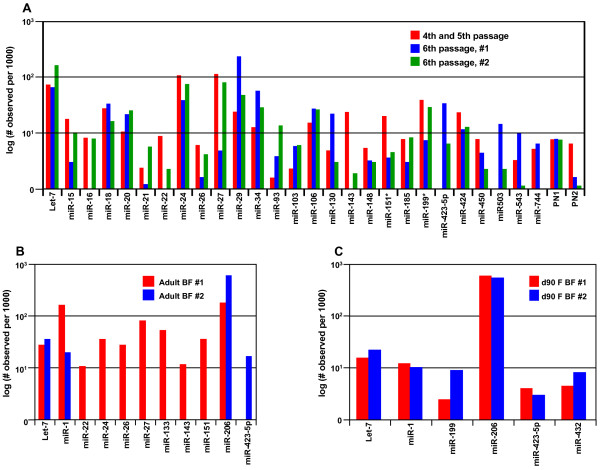
**Variation between microRNA libraries**. Transcriptome profiles from samples at the same developmental state were compared within the three satellite cell libraries (2a), two individual adult biceps femoris libraries (2b), and two d 90 fetal biceps femoris libraries (2c). MicroRNA cDNA libraries for satellite cells (2a) were created from the pool of the 4th and 5th passage satellite cells and the 6th passage (6th passage, #1). A second library was created from a second flask of 6th passage stellite cells (6th passage, #2) to evaluate variation between satellite cells at the same passage. MicroRNA abundance is represented as number of individual miR tags observed per thousand tags evaluated.

Comparison of miR expression profiles between libraries created from independent samples at the same developmental state was of interest to examine biological and technical replication of the clone-based approach to miR profiling. Previous studies in other species have not specifically addressed this issue. Therefore, two approaches were completed to evaluate possible variation between miR libraries. First, transcriptome profiles were compared between miR libraries that were replicated in the current experiment including the three proliferating satellite cell, two adult biceps femoris, and two d 90 fetal biceps femoris libraries (Figure 2). As detailed in the Methods, RNA was obtained from multiple cell culture isolations of proliferating satellite cells at different cell passages including 4^th^, 5^th^, and 6^th ^passages. The 4^th ^and 5^th ^passages were pooled together and compared to two different cell culture isolations from the 6^th ^passage cells. The miR trascriptome profiles from the two 6^th ^passage samples differed from each other as much as from the 4^th ^and 5^th ^passage sample (Figure 2a). Therefore, variability in abundance was quantified using a histogram (data not shown) of relative changes in abundance. Data utilized in the histogram was restricted to miR observed at a minimum abundance ratio level of 5 per thousand miR observed. This analysis resulted in 30 observed changes between samples that were not expected to have differing transcriptome profiles. Of these observed changes, the majority of changes were small (17 of the 30 ratios were between 1 and 2 per thousand miR observed, with an additional 8 between 2 and 3 per thousand miR observed). However, four ratios (13%) were between 3 and 6 per thousand miR expressed, with one almost reaching a ratio of 10. As a result of this analysis, we concluded that changes in abundance are likely to be significant if they are greater than 6-fold. Upon final evaluation of the miR transcriptome profiles, we took a more conservative approach and restricted our discussion to changes in miR abundance levels that were 10-fold or greater. Second, two individual libraries from the same 6th passage RNA ligation template were created to determine if variability was introduced during the PCR amplification step (see Methods). The transcriptome profiles of the replicate libraries were identical (data not shown), suggesting that variation observed previously between the three satellite cell libraries was due to satellite cell populations.

Further evaluation of miR tags from the adult biceps femoris libraries (Figure 2b) demonstrated reproducible abundance levels between libraries of the moderate to highly abundant miR including let-7 and the muscle-specific miR-1 and miR-206. However, clear differences between the two libraries were evident as seven additional miR were identified in the first library compared to the second. In contrast to the adult and satellite cell miR libraries, the fetal libraries exhibited similarity across all miR that were present (Figure 2c). This observed variation in miR abundance within the adult libraries versus the fetal libraries could be attributed to animal variation. For the fetal libraries, biceps femoris of four female fetuses was collected and pooled for RNA extraction separately from two individual sows. The pooling strategy necessary to obtain sufficient starting material for the fetal muscle samples may have reduced variation present between individual samples and present a less variable overall miR profile. In contrast, each adult library was created from a muscle sample of an individual sow. Additionally, difference between libraries could be a result of variation in muscle sample. Muscle is not a homogeneous tissue [31], and it has been reported that gene abundance levels change based on location of the sample and distribution of Type I and Type II muscle fibers throughout the sample [32-36]. For the fetal libraries, the entire biceps femoris was obtained from four fetuses. The pooled sample was then powdered to a homogenous mix for RNA extraction. However, for the adult libraries a two to four gram sample was obtained for RNA extraction due to the greater size of the adult muscle. Therefore, it is possible that the two biceps femoris samples for the adult libraries were heterogeneous and may have contributed to the variation in miR tags between adult libraries.

### MicroRNA transcriptome profiles in skeletal muscle

A digital transcriptome profile approach [37] was applied to evaluate miR abundance based on cloning the miR population from each sample and evaluating abundance as the number of transcripts for a given miR gene per thousand transcripts observed (see Additional file 1). This cloning and sequencing-based approach was highly successful, as indicated by the high degree of homology between our results and those previously described in miRbase for other species.

**Figure 3:**
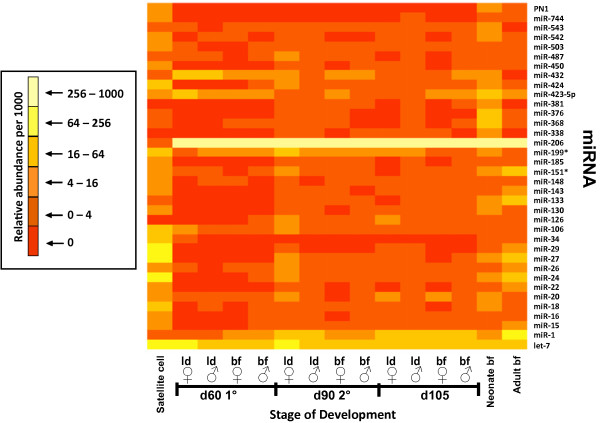
**MicroRNA transcriptome profiles**. MicroRNA cDNA clone libraries were created from skeletal muscle during specific stages of swine development including satellite cells, d 60 (primary fiber development) of fetal development, d 90 (secondary fiber development) of fetal development, d 105 of fetal development, one day-old neonate and the adult. For the fetal samples, biceps femoris (BF) and longissimus dorsi (LD) were collected from female and male fetuses. Biceps femoris (BF) samples were collected for the one day-old neonate and the adult. MicroRNA abundance is represented as number of individual miR tags observed per thousand tags evaluated. Data for the satellite cells and adult muscle is presented as the average of multiple transcriptome libraries presented in Additional file 1.

Sequence comparison to known miR identified the muscle-specific miR-206 as the highest abundant miR across all muscle samples, which represented greater than 60% of all miR present (Figure 3). This contrasts sharply with the abundance of miR-206 in proliferating satellite cells at 1.8 per thousand (or 0.18%). In mouse C_2_C_12 _cells, miR-206 is also lowly abundant in proliferating cells and has been reported to be induced during differentiation [38], suggesting that its presence is associated with the switch from precursor to mature muscle cell. In addition, mR-1 and miR-133 are lowly abundant in proliferating C_2_C_12 _myoblasts [38]. The data herein confirm these results in satellite cells and also demonstrate that miR-206 is present at a high level through most or all of fetal development, and continues through maturation of the adult pig. The constant high-level presence at all stages following early differentiation suggests that the role of miR-206 is to repress functions associated with muscle precursor cells.

In comparison to the high abundance of miR-206 in muscle tissue, porcine miR-1 had relatively moderate abundance that increased throughout development, similar to the pattern observed for this miR in mouse muscle development [16]. These data suggest that miR-1 and miR-206 play different roles in muscle development, with miR-1 affecting regulation of genes that require inactivation in later fetal stages and miR-206 having a more constant role in repressing genes immediately after differentiation. In contrast to miR-1 and miR-206, miR-133 was detected only at low levels in fetal development yet increased in the neonate and adult (Figure 3). Based on abundance level, these data suggest that miR-206 and miR-1 may have a greater role in fetal muscle development than miR-133, or their targets are higher in abundance. In addition, both miR-206 and miR-1 promote differentiation [16], suggesting that these two miR may have a greater impact compared to miR-133 on increased differentiation, which characterizes fetal development [39].

In addition to muscle-specific miR, a larger number of ubiquitous miR were present across all libraries. While a greater percentage of ubiquitous miR were lowly abundant throughout development, a number increased in abundance at specific developmental stages. MiR present 10 fold greater in satellite cells compared to neonate muscle included miR-16, miR-18, miR-27, miR-29, miR-34, and miR-106. Slightly below the factor-of-ten cut-off included miR-24 at 9.2. These miR have been implicated in multiple cellular processes including cell growth (miR-24), apoptosis (miR-16, miR-24 and miR-29), and cell cycle regulation in normal (miR-16) and cancerous cells (miR-24, miR-27, miR-29 and miR-34) [9,40-46]. Conversely, research has reported low abundance during proliferation and differentiation in cell culture for these miR with the exception of miR-24, which has a moderately high abundance level during late differentiation [16]. This difference in abundance level may be due in part to the different cell culture models utilized in the experiments. Chen et al. [16] evaluated miR in an immortalized C_2_C_12 _cell line, while the experiment herein utilized a porcine primary muscle cell line (satellite cells) during proliferation.

The fetal time points examined in swine were selected to coincide with important events in muscle development, specifically the waves of primary and secondary fiber formation (See Methods). Overall, miR were lowly abundant throughout fetal development with the exception of let-7 and muscle specific miR-1 and miR-206. Differential abundance of lowly to moderately abundant miR was observed between time points for primary (d 60) and secondary fiber development (d 90 and d 105; Figure 3). MiR-432 was moderately abundant during early fetal development at d 60, while miR-424 abundance increased during d 90 and d 105, and miR-126 abundance increased during later stages of fetal development at d 90 and 105. Currently, function of miR-432 in muscle development has not been determined. However, previous research reports that miR-424 regulates monocyte and macrophage differentiation [47], while miR-126 expression alters cell cycle progression of cancer cells by decreasing tumor growth and proliferation [48]. Four miR, miR-338, miR-368, miR-376, and miR-381, were also identified to be specific to the one-day old neonate as demonstrated by a ten-fold increase in abundance level, compared to both the adult muscle and satellite cells, suggesting that these miR may have a role in muscle growth immediately following birth. As for the adult, miR abundance was greatest for miR-151 and the muscle specific miR-1 and miR-133 compared to fetal and neonate miR libraries. While miR-1 and miR-133 abundance increases during differentiation in cell culture [16], the role of these miR in adult skeletal muscle has yet to be fully determined.

### Classification of potential novel miR

Evaluation of sequence clusters identified two different classifications of novel miR; sequence tags that differed at only one (highly conserved) position and sequence tags that had no match to miR in the database. Five observed tags, miR-168a, miR-206, miR-24a, miR-368, and miR-381, represented possible exceptions to the pattern of exact sequence conservation across positions 4–17 of the reference miR. These five observed sequences differed from known miR at only one position, and the miR were observed between 23 and 59 times, and are not likely to be attributable to experimental artifacts. Three of the five were observed more frequently without mismatches: miR-206, miR-24a, and miR-168a (a cross-contamination from the parallel control oligo processing, see Methods). Interestingly, all of the miR-381 related tags displayed mismatches with the reported human miR-381 sequence with a single mismatch occurring 59 times (G?A at position 10 in *Sus scrofa *miR sequence), indicating that the *Sus scrofa *version of miR-381 does indeed differ in sequence from human and mouse. MiR-368 was observed 23 times as a mismatch and only 12 times without a mismatch. These five examples of single base mismatches were included in the transcriptome profiles along with those that matched exactly.

In addition to identification of mismatches in the miR-206 sequence, length of sequence at the 5' and 3' ends also differed (Table 1, see Additional file 2). While there are known instances of highly similar or identical mature miR being produced from discrete genes, it seems more likely that these differences are attributed to minor alterations in processing, cloning or sequencing of miR originating from the same gene. Since the goal was to produce relative transcriptome profiles of potential miR during muscle development, we clustered sequences of this type into a single miR sequence, using the most commonly observed sequence of the cluster as the defining sequence for comparisons to known miR.

**Table 1:** miR-206 sequence variation

**Sequence**	**Quantity observed**
UGGAAUGUAAGGAAGUGUGUGA	2286

UGGAAUGUAAGGAAGUGUGUGAA	79

UGGAAUGUAAGGAAGUGUGUGU	78

UGGAAUGUAAGGAAGUGUGU	41

UGGAAUGUAAGGAAGUGUGUGAU	34

UGGAAUGUAAGGAAGUGUGUGA	15

UGGAAUGUAAGGAAGUGUGUG	13

UGGAAUGUAAGGAAGUGUGU	9

UGGAAUGUAAGGAAGUGUGUGG	8

CGGAAUGUAAGGAAGUGUGUGA	7

Mir-206 differed in length or sequence at the 5' and 3' ends. MiR-206 sequences of this type were clustered into a single miR sequence identified as the predominant sequence. Mir-206 sequences with the greatest observations are listed.

Combining the data from all transcriptome profiles identified a total of 94 distinct miR that matched reference sequences and 12 sequences that did not match, and are therefore potential novel or porcine-specific miR (given temporary identifiers PN (porcine new) 1 to PN12; Table 2). While a greater number of these novel miR were lowly abundant throughout development, PN1 abundance increased in the proliferating satellite cells, neonate, and adult. Further validation of these putative novel miR awaits development of the swine genome to look for hallmarks of microRNA genes related to these tag sequences.

**Table 2:** Novel porcine miR

**Temporary identifiers**	**Sequence**
PN1	CCGCAGGUGCGGCCACUUGUUU

PN2	GUGUUGGUGUGCACUUAUUU

PN3	CGAACCGAAUCCCUCACUAAA

PN4	AGGGGAGUGGUGGGGGGAG

PN5	CGAACCGAACUCCUCACUAAA

PN6	AGGGUUGGGCGGAGGCUUUCC

PN7	CCACGAGGAGGAGACGCAGUG

PN8	UGGCACAGGGUCCAGCUGUCGGC

PN9	GGGGUGGGGGUCUGGGGGGUGU

PN10	GAGAGAUCAGAGGCGCAGAGU

PN11	GUGUGGGACGGUGGGGUGGGUU

PN12	GUCGGGGAGGUUCCAGCUCUCAUUU

Twelve novel miR were detected that were not closely related to previously reported miR in the database. The miR were given temporary identifiers PN (porcine new) 1 to PN12.

### Computational identification of miR targets

Relatively few miRNA targets have been identified experimentally, but numerous computational predictions are readily available including miRanda, RNAhybrid and TargetScan [49-51]. Initially, miR targets were predicted for a sub-set of the miR up-regulated in this experiment (miR-206, miR-338, miR-368, miR-376a, and miR-381). These miR were predicted by miRNA viewer to target 47 "common genes" and 864 genes when the "all genes" option was chosen. For the purposes of this study, the predicted target genes were narrowed down to include only those with "muscle" listed in the gene ontology, as noted by the Entrezgene project [52]. A total of 19 genes were selected based on these criteria (see Additional file 3). Of these, 7 targets were identified for the highest expressed miR during fetal development to the adulthood, miR-206, including genes that have been implicated in multiple myogenic processes (see Additional file 3) [50,53-56]. These predicted targets for miR-206 include dystophia myotonica protein kinase, which has been implicated in myotonic dystrophy [55] and the transcription factor paired box gene 3 that regulates myogenic cell fate through the myogenic transcription factor MyoD [54]. Secondly, targets for a sub-set of down-regulated miR (miR-15, miR-16, miR-27, miR-29, miR-34 and miR-106), of which 44 targets were identified based on our previous criteria were predicted (see Additional file 4). From these identified gene targets, the ability to link the specific miR to target transcripts will improve as computational methods evolve, and as the databases of expressed and genomic porcine sequences grow.

## Conclusion

Together, these data suggest that miR have a role in progression of myogenesis throughout development and their function may be specific to different stages of skeletal muscle growth. In addition, the data reported herein are the most complete set of transcriptome profiles to evaluate miR abundance in skeletal muscle at specific time points during fetal development of swine. Identification of these miR provide an initial group of expressed miR that change in abundance during specific developmental stages and therefore may target genes that regulate this process.

## Methods

### Skeletal muscle collection and clone libraries

MicroRNA libraries for the satellite cells were created from cells cultured from semimebranosus of 8-week old piglets as previously described [57] and incubated at 37°C with 5% CO_2_. Satellite cells at passage four, five, and six were collected for RNA extraction. RNA from the 4^th ^and 5^th ^passages was combined for creation of the first satellite cell cDNA library. RNA from two sets of 6^th ^passage satellite cells were used for the second cDNA library. Tissues during fetal development were collected at d 60, 90, and 105 of fetal development [57] by removing porcine fetuses immediately after sacrifice and dissecting longissimus dorsi and biceps femoris muscle. Four female and four male fetuses were obtained from a single sow at each time point, and samples were pooled by sex and muscle type before immersion in liquid nitrogen. Neonatal biceps femoris was obtained at day one after birth, while two to four grams of adult biceps femoris was also obtained from an adult sow at slaughter. The experimental procedures were approved and performed in accordance with U. S. Meat Animal Research Center Animal Care Guidelines and the Guide for the Care and Use of Agricultural Animals in Agricultural Research and Teaching (1999). RNA extraction was performed using TRIreagent following the manufacturer's recommended protocol (Ambion, Austin, TX). Concentration and quality of RNA was determined using an Agilent 2100 Bioanalyzer for RNA (Agilent Technologies, Santa Clara, CA). Single insert cDNA libraries were constructed as described previously by Lu et al. [58] with the following modifications. RNA fractions were isolated from denaturing acrylamide gels and eluted by FlashPAGE (Ambion, Austin, TX). First strand cDNA synthesis was performed utilizing a primer to the 3' adapter sequence and SUPERSCRIPT reverse transcriptase (Invitrogen, Carlsbad, CA). The microRNA were then amplified by PCR and digested with EcoRI for ligation into pBLUEscript and electroporation in EC100 electrocompetent cells. Individual colonies were transferred into 384-well plates and grown in ampicillin selective LB media for plasmid preparation and sequencing using an Applied Biotechnology 3730 sequencer.

### Statistical analysis

Chromatograms were converted into sequences and scored using Phred [59]. Sequences were collected based on identification of flanking vector and linker sequences. The intervening sequences were kept as putative miR, as long as their length was between 16 and 27 bases. The putative miR were then clustered based on sequence similarity into characteristic consensus sequences, where each member was required to match 14 consecutive bases to the most common member of the cluster. This approach was used due to frequent observance of highly similar miR differing only at their 3' ends, which often varied only by length or in the sequence of the last base [27]. The characteristic sequences were then compared to all known miR from the miRBase [21-23]. The criterion used for a positive match was that the putative miR contained an exact match to positions 4–17 of a known miR, as this segment of the miR sequence are highly conserved and unique to each miR. Validity of our clustering approach was tested using 455 known human miR. This resulted in 401 miR matching only to their unique sequence. Of the remaining 54 sequences, none involved the human homologue of the porcine miR reported in our current experiment. While it is possible that a portion of the clusters may represent miR from more than one distinct gene, the homology of the core targeting sequence indicates they are likely to have similar targets. Those that were not identified this way were screened, using BLAST, against tRNA, rRNA, snoRNA and mitochondrial sequence. The remaining unidentified sequences were checked for single base mismatches within positions 4–17 of previously identified miR. Only five examples were found, and they were counted with the full matching sequences. The observed putative miR that had at least two mismatches and that were observed at least 20 times were given temporary labels PN (porcine new) 1 to PN12 in order of decreasing levels of abundance.

MicroRNA were considered expressed if the tag cluster had at least ten members per thousand tags observed. Low abundance was defined as 0 to 16 tags per thousand observed, moderate abundance was defined as 16 to 256 tags, and high abundance was defined as greater than 256 tags (Figure 3). A difference between tag counts was accounted if greater than 10 fold.

## Abbreviations

miR: microRNA; MiRNA: microRNA; mRNA: messenger RNA; PCR: polymerase chain reaction; PN: Porcine new; RISC: RNA induced silencing complex; RNA: ribonucleic acid

## Authors' contributions

TGM conducted experimental design, tissue collection, RNA extraction, cDNA library construction, and participated in drafting of the manuscript. TPLS conceived the project and participated in experimental design and drafting of the manuscript. TPLS, LLC and TSD participated in training for development of cDNA libraries. MED collected and provided satellite cells. JRM participated in experimental design and tissue collection. LKM participated in data analysis. DJN provided tissue for day-old neonate. RTW conducted data analysis and participated in drafting the manuscript. All authors read and approved of the final manuscript.

## Supplementary Material

Additional file 1 **Adundance levels of miR in skeletal muscle at specific developmental states.** The data provided represent the transcription profiles of miR during specific stages throughout skeletal muscle development in swine. Click here for file

Additional file 2 **miR-206 sequence variation. The data provided represent the variation of miR-206 sequences identified in the current study.** Click here for file

Additional file 3 **Predicted targets of up-regulated miR.** The data provided represent the predicted gene targets for the up-regulated miR. Click here for file

Additional file 4 **Predicted targets of up-regulated miR.** The data provided represent the predicted gene targets for the down-regulated miR. Click here for file
