# Supplementary material for: MicroRNA transcriptome profiles during swine skeletal muscle development
Source: BMC Genomics. 2009 Feb 10;10:77. doi: 10.1186/1471-2164-10-77 (PMC2646747; doi:10.1186/1471-2164-10-77)
Supplement: Additional file 1 — Adundance levels of miR in skeletal muscle at specific developmental states. The data provided represent the transcription profiles of miR during specific stages throughout skeletal muscle development in swine. [file 1471-2164-10-77-S1.docx]

### Additional file 1. Abundance levels of miR in skeletal muscle at specific developmental states in swine.

miR Name Satelllite 4th and 5th 6th 1st run 6th 2nd run F LD d60 M LD d60 F BF d60 M BF d60 F LD d90 M LD d90 F BF d90 F BF d90 F BF d90 M BF d90 F LD d105 M LD d105 F BF d105 M BF d105 Neonate Adult F BF Adult F BF Adult F BF

Cell Average Average 1^st^ run 2^nd^ run Average 1^st^ run 2^nd^ run

let-7 84.98 74.33 66.54 165.20 65.28 26.69 27.11 33.55 67.62 41.94 19.54 16.21 23.32 39.75 48.65 38.97 40.11 32.72 40.94 27.38 25.53 31.66

miR-1 1.60 2.67 N/D N/D 2.37 0.76 15.63 11.18 30.11 17.76 9.57 10.81 8.16 7.31 29.07 31.13 30.47 17.20 12.15 100.22 138.85 18.54

miR-10 N/D 0.09 N/D N/D N/D N/D N/D N/D N/D 0.12 N/D N/D N/D N/D N/D N/D N/D N/D N/D N/D N/D N/D

miR-15 13.00 18.00 3.04 10.30 N/D N/D 0.64 N/D 2.32 0.74 0.27 0.26 0.29 0.46 0.86 0.23 0.39 0.13 0.21 5.16 7.60 N/D

miR-16 6.18 8.29 0.41 8.01 N/D N/D 0.64 N/D 1.16 0.62 N/D N/D N/D 0.23 0.58 0.12 0.77 0.13 1.40 0.72 1.06 N/D

miR-18 27.87 27.99 33.68 16.41 N/D 0.25 0.96 N/D 1.62 1.11 0.27 N/D 0.58 0.23 0.58 0.82 N/D 0.91 9.38 2.44 3.59 N/D

miR-20 15.72 10.70 21.91 25.56 2.84 1.01 1.59 0.86 4.17 0.86 N/D N/D N/D 2.51 4.32 1.40 5.01 1.42 4.48 1.29 1.69 0.45

miR-21 2.56 2.41 1.22 5.72 N/D N/D N/D N/D N/D N/D 0.14 N/D 0.29 0.69 N/D 0.23 N/D 0.13 N/D N/D N/D N/D

miR-22 5.75 8.91 0.41 2.29 N/D N/D N/D N/D 0.23 0.25 N/D N/D N/D 0.23 N/D 0.35 N/D 0.26 2.13 9.61 14.14 N/D

miR-23 1.92 2.32 0.61 2.67 N/D N/D N/D N/D N/D 0.25 N/D N/D N/D N/D N/D 0.12 N/D N/D 1.49 0.57 0.84 N/D

miR-24 86.53 109.36 39.16 75.93 N/D N/D 0.96 N/D 4.17 0.99 0.68 0.51 0.87 0.23 0.29 2.22 1.54 3.62 9.59 25.09 36.93 N/D

miR-26 4.69 6.15 1.62 4.20 0.47 N/D 0.96 0.43 6.25 1.60 0.96 1.03 0.87 0.69 0.29 2.69 0.77 1.03 3.20 12.33 18.15 N/D

miR-27 81.73 115.42 4.87 81.65 N/D N/D N/D N/D 5.56 1.23 1.37 1.29 1.46 0.46 1.73 1.76 N/D 0.91 4.69 60.22 88.63 N/D

miR-29 83.81 24.24 238.18 48.45 N/D N/D N/D N/D 0.23 N/D N/D N/D N/D N/D 0.29 0.12 N/D N/D 2.77 5.73 8.44 N/D

miR-30 0.96 0.80 1.22 1.14 N/D N/D N/D N/D N/D 0.25 N/D N/D N/D N/D 0.29 0.47 0.77 0.65 1.71 1.43 1.90 0.45

miR-34 26.80 12.83 57.42 29.00 0.47 N/D N/D N/D N/D N/D N/D N/D N/D N/D N/D N/D N/D N/D 1.07 0.29 0.42 N/D

miR-92 0.43 0.45 0.61 N/D N/D N/D N/D N/D N/D N/D N/D N/D N/D N/D 0.29 N/D N/D N/D N/D N/D N/D N/D

miR-93 3.89 1.60 3.85 13.74 3.31 1.51 0.96 3.01 1.85 0.74 0.27 0.26 0.29 0.69 1.44 0.47 1.93 0.91 N/D 0.86 0.63 1.36

miR-95 N/D N/D N/D N/D N/D N/D N/D N/D 0.23 N/D N/D N/D N/D N/D N/D N/D N/D N/D N/D N/D N/D N/D

miR-98 1.28 1.07 1.22 2.29 0.47 N/D N/D N/D N/D 0.12 N/D N/D N/D 0.46 0.29 0.23 0.39 N/D N/D N/D N/D N/D

miR-101 0.32 0.27 0.20 0.76 N/D N/D N/D N/D N/D 0.12 N/D N/D N/D N/D 0.29 0.23 N/D N/D N/D 2.15 3.17 N/D

miR-103 3.78 2.32 5.88 6.10 N/D 1.51 0.64 N/D 0.93 0.37 0.82 1.54 N/D N/D N/D 0.35 1.93 0.52 2.56 0.43 0.21 0.90

miR-106 20.25 15.51 27.59 26.71 5.20 1.51 2.23 3.01 5.56 1.48 1.23 1.29 1.17 2.06 0.58 1.99 1.54 2.20 2.13 2.29 2.95 0.90

miR-122 0.11 0.18 N/D N/D N/D 0.76 N/D N/D N/D N/D N/D N/D N/D 0.46 N/D N/D N/D 0.13 N/D 0.29 N/D 0.90

miR-126 N/D N/D N/D N/D N/D N/D 0.64 N/D 4.17 1.73 0.41 0.26 0.58 1.60 4.03 1.29 3.47 1.55 1.28 2.29 3.38 N/D

miR-126* N/D N/D N/D N/D N/D N/D N/D N/D N/D N/D N/D N/D N/D N/D N/D 0.12 N/D N/D N/D 0.72 1.06 N/D

miR-127 N/D N/D N/D N/D N/D N/D N/D N/D 0.23 0.37 N/D N/D N/D 0.46 0.29 0.23 N/D N/D N/D N/D N/D N/D

miR-128 0.16 0.18 N/D N/D N/D 0.25 N/D N/D 0.23 0.37 0.27 N/D 0.58 N/D N/D 0.59 N/D 0.39 N/D 0.86 1.27 N/D

miR-130 9.22 4.90 22.32 3.05 N/D N/D N/D N/D 1.85 1.23 N/D N/D N/D 0.46 1.44 0.82 1.54 0.26 6.40 0.29 0.42 N/D

miR-133 0.27 0.45 N/D N/D N/D N/D N/D N/D 0.69 0.12 0.27 N/D 0.58 0.46 N/D 0.47 1.16 0.26 4.05 31.68 46.63 N/D

miR-136 N/D N/D N/D N/D 0.47 N/D N/D 0.43 0.46 0.37 N/D N/D N/D 0.91 2.01 1.40 0.77 0.52 1.28 N/D N/D N/D

miR-139 N/D N/D N/D N/D N/D N/D 0.32 N/D N/D N/D 0.14 0.26 N/D N/D N/D N/D N/D N/D N/D 0.29 0.42 N/D

miR-140 1.23 1.78 N/D 1.14 N/D N/D N/D N/D 0.23 N/D N/D N/D N/D N/D N/D N/D N/D N/D N/D 1.29 1.90 N/D

miR-143 14.81 24.06 0.61 1.91 N/D N/D N/D N/D 1.85 0.62 0.27 N/D 0.58 N/D 0.29 0.59 0.39 0.65 2.56 11.33 16.67 N/D

miR-148 4.53 5.44 3.25 3.05 N/D 0.50 N/D 0.43 2.32 N/D 0.14 N/D 0.29 0.46 0.58 1.05 0.39 0.26 1.92 1.00 1.48 N/D

miR-149 N/D N/D N/D N/D N/D N/D N/D N/D N/D N/D N/D N/D N/D N/D N/D N/D N/D 0.13 N/D N/D N/D N/D

miR-151* 13.75 20.32 3.65 4.58 0.47 0.25 0.96 N/D 6.95 1.48 1.09 1.03 1.17 1.37 N/D 1.40 1.16 1.42 4.48 21.79 31.65 0.90

miR-152 3.14 4.01 1.62 2.29 N/D N/D N/D N/D 0.69 N/D 0.14 N/D 0.29 N/D N/D 0.23 N/D N/D 0.21 0.14 0.21 N/D

miR-155 0.32 0.09 N/D 1.91 N/D 0.25 N/D N/D 0.23 N/D N/D N/D N/D N/D N/D N/D N/D N/D N/D N/D N/D N/D

miR-168 N/D N/D N/D N/D N/D 0.50 N/D 2.58 N/D N/D N/D N/D N/D N/D N/D N/D N/D N/D N/D N/D N/D N/D

miR-181 0.91 0.09 2.64 1.14 1.42 1.01 N/D N/D 3.94 1.60 0.27 0.26 0.29 1.14 3.74 2.93 2.31 0.78 2.56 1.43 1.69 0.90

miR-184 N/D N/D N/D N/D N/D N/D N/D N/D N/D N/D N/D N/D N/D N/D N/D 0.12 N/D N/D N/D N/D N/D N/D

miR-185 6.66 7.84 3.04 8.39 N/D N/D N/D N/D 1.62 0.37 0.14 0.26 N/D 0.23 N/D 0.82 N/D 0.91 2.13 0.14 0.21 N/D

miR-191 1.39 0.98 2.64 0.76 N/D N/D 0.64 N/D 0.23 N/D N/D N/D N/D 0.23 N/D 0.12 N/D 0.13 1.28 N/D N/D N/D

miR-193* 1.60 0.09 0.20 0.38 N/D N/D N/D N/D 0.69 N/D N/D N/D N/D N/D 0.29 N/D N/D N/D 0.21 0.14 0.21 N/D

miR-195 1.01 1.43 N/D 1.14 N/D N/D N/D N/D N/D N/D 0.14 0.26 N/D 0.23 0.29 0.35 0.39 0.13 0.21 0.14 0.21 N/D

miR-196 0.37 0.27 0.20 1.14 N/D N/D 0.32 N/D 0.23 0.12 N/D N/D N/D N/D 0.29 N/D 0.39 0.39 N/D 0.14 0.21 N/D

miR-199 0.32 0.53 N/D N/D N/D N/D N/D N/D N/D N/D N/D N/D N/D N/D N/D N/D 0.39 N/D N/D N/D N/D N/D

miR-199* 29.68 39.48 7.51 29.38 3.31 4.03 12.76 7.31 35.90 10.98 5.60 2.32 9.33 8.68 7.20 11.23 3.47 3.62 11.94 3.30 4.64 0.45

miR-206 1.76 1.25 1.83 3.82 692.05 827.29 822.01 827.96 606.99 800.52 872.76 886.54 857.14 808.54 737.48 779.43 767.84 832.79 565.67 363.44 155.73 812.75

miR-210 N/D 0.09 0.41 0.76 N/D N/D N/D 0.43 0.46 N/D N/D N/D N/D N/D N/D N/D N/D N/D N/D N/D N/D N/D

miR-214 2.18 2.23 1.83 2.67 0.95 0.25 1.28 N/D N/D N/D 0.41 0.26 0.58 0.23 N/D 0.12 0.39 0.26 0.43 0.29 0.42 N/D

miR-320 0.11 N/D 0.41 N/D N/D N/D N/D N/D 0.46 N/D 0.14 0.26 N/D 0.23 1.44 0.35 0.77 N/D N/D N/D N/D N/D

miR-324 0.59 0.36 1.22 0.38 N/D N/D N/D N/D 0.46 N/D 0.41 0.51 0.29 0.23 0.29 0.23 N/D 0.26 2.35 0.14 0.21 N/D

miR-335 N/D 0.09 N/D N/D N/D N/D N/D N/D N/D N/D N/D N/D N/D 0.46 N/D N/D N/D N/D N/D N/D N/D N/D

miR-338 N/D N/D N/D N/D N/D N/D N/D N/D 0.23 0.12 N/D N/D N/D 0.69 0.86 0.82 0.39 N/D 6.18 N/D N/D N/D

miR-352 N/D N/D N/D N/D N/D N/D N/D N/D N/D N/D N/D N/D N/D 0.23 N/D N/D N/D N/D N/D N/D N/D N/D

miR-361 0.21 0.27 0.20 N/D N/D N/D N/D N/D 0.23 N/D N/D N/D N/D N/D N/D N/D N/D N/D N/D 0.29 0.42 N/D

miR-363 0.21 0.18 0.20 0.38 N/D N/D N/D N/D N/D N/D N/D N/D N/D N/D N/D N/D 0.39 N/D N/D N/D N/D N/D

miR-368 0.80 0.80 1.01 0.38 N/D N/D 0.32 0.43 2.78 0.49 0.96 0.51 1.46 N/D N/D 0.94 N/D 0.78 27.29 0.57 0.84 N/D

miR-369 N/D N/D N/D N/D N/D N/D N/D N/D 1.39 0.62 N/D N/D N/D N/D N/D 0.35 N/D N/D N/D N/D N/D N/D

miR-370 N/D N/D N/D N/D N/D N/D N/D N/D 0.23 N/D N/D N/D N/D 0.23 N/D N/D N/D N/D N/D N/D N/D N/D

miR-374 0.59 0.98 N/D N/D N/D N/D N/D N/D 0.23 0.12 N/D N/D N/D N/D N/D N/D N/D N/D N/D N/D N/D N/D

miR-376 0.85 0.71 1.62 N/D N/D N/D N/D N/D 0.23 0.62 0.27 0.51 N/D N/D N/D 0.47 N/D N/D 21.75 0.29 0.42 N/D

miR-380 N/D N/D N/D N/D N/D N/D N/D N/D 0.46 0.12 N/D N/D N/D N/D N/D N/D N/D 0.13 N/D N/D N/D N/D

miR-381 N/D N/D N/D N/D N/D N/D N/D N/D 0.46 0.37 0.41 0.26 0.58 0.23 N/D 0.47 N/D 0.13 12.58 N/D N/D N/D

miR-382 N/D N/D N/D N/D N/D N/D N/D 0.86 N/D 0.25 N/D N/D N/D 0.46 N/D 0.23 N/D N/D N/D N/D N/D N/D

miR-409 N/D 0.18 N/D N/D 2.37 N/D 3.83 0.86 3.94 1.11 0.27 0.26 0.29 1.83 0.86 1.05 1.54 0.39 N/D N/D N/D N/D

miR-411 0.11 0.09 0.20 N/D N/D N/D N/D N/D 0.23 N/D N/D N/D N/D N/D N/D N/D N/D N/D 1.71 N/D N/D N/D

miR-422 N/D 0.09 0.20 N/D N/D N/D N/D N/D 0.46 0.12 N/D N/D N/D N/D N/D 0.12 N/D N/D N/D 0.72 1.06 N/D

miR-423-5p 13.00 N/D 34.29 6.49 18.92 14.10 7.66 5.59 3.94 2.47 4.10 5.15 2.92 3.43 1.44 1.40 6.17 4.66 19.40 5.02 0.21 14.93

miR-424 19.07 23.71 11.77 12.97 N/D N/D 0.32 N/D 9.03 2.34 1.64 1.29 2.04 0.23 0.58 1.29 0.77 0.78 4.48 3.01 4.43 N/D

miR-425 0.37 0.45 0.20 0.38 N/D N/D N/D N/D 0.23 0.12 N/D N/D N/D N/D N/D N/D N/D N/D N/D 0.72 1.06 N/D

miR-431 N/D N/D N/D N/D N/D N/D N/D N/D 1.39 0.37 0.55 0.26 0.87 0.46 0.29 0.70 N/D 0.65 N/D N/D N/D N/D

miR-432 0.27 0.36 N/D 0.38 25.07 37.01 13.40 8.17 8.11 2.34 7.52 5.66 9.62 6.85 1.15 2.34 1.54 4.78 4.48 N/D N/D N/D

miR-449 N/D N/D 0.41 N/D N/D N/D N/D N/D 0.23 N/D N/D N/D N/D N/D N/D N/D N/D N/D N/D N/D N/D N/D

miR-450 6.18 7.84 4.46 2.29 N/D N/D N/D N/D 3.94 1.11 N/D N/D N/D 0.23 0.58 0.94 N/D 0.26 2.35 2.29 3.38 N/D

miR-455* 0.32 0.18 N/D 1.53 N/D N/D N/D N/D N/D N/D 0.82 N/D 1.75 N/D N/D 0.12 N/D N/D N/D 0.29 0.42 N/D

miR-483 N/D N/D N/D N/D N/D N/D N/D N/D 0.23 N/D N/D N/D N/D 0.23 N/D N/D N/D N/D N/D N/D N/D N/D

miR-484 N/D 0.62 0.41 N/D N/D N/D N/D N/D 0.23 N/D N/D N/D N/D N/D N/D N/D N/D N/D N/D N/D N/D N/D

miR-487 0.59 0.80 0.20 0.38 0.47 0.76 2.23 N/D 7.41 2.10 1.09 0.77 1.46 0.46 N/D 1.99 N/D 1.03 1.71 0.29 0.42 N/D

miR-493 N/D N/D N/D N/D 1.89 1.51 1.28 0.43 1.16 0.62 0.55 0.77 0.29 1.60 0.29 0.35 2.70 0.13 N/D N/D N/D N/D

miR-495 0.11 0.18 N/D N/D N/D 0.50 1.59 0.43 1.39 0.25 0.27 0.26 0.29 0.91 0.29 0.47 N/D 0.52 1.92 N/D N/D N/D

miR-497 1.23 1.43 1.22 0.38 N/D N/D N/D N/D N/D 0.12 N/D N/D N/D N/D N/D N/D N/D N/D 0.64 0.72 1.06 N/D

miR-499 0.11 0.09 N/D 0.38 N/D N/D 0.32 N/D 0.23 N/D N/D N/D N/D N/D 0.29 0.35 0.39 N/D N/D 2.72 4.01 N/D

miR-500 N/D N/D N/D N/D N/D N/D N/D N/D 0.23 N/D N/D N/D N/D N/D N/D N/D N/D N/D N/D N/D N/D N/D

miR-503 4.74 0.98 14.61 2.29 0.47 N/D 0.64 N/D 2.08 0.99 0.14 N/D 0.29 0.69 2.30 0.12 0.39 0.13 1.92 0.57 0.84 N/D

miR-532 0.16 0.27 N/D N/D N/D N/D 0.32 N/D 0.23 0.12 N/D N/D N/D N/D N/D 0.12 N/D 0.13 N/D N/D N/D N/D

miR-542 4.74 3.30 9.94 1.14 N/D 0.25 N/D 0.43 1.16 0.49 N/D N/D N/D N/D 0.29 0.35 N/D 0.78 12.79 0.57 0.84 N/D

miR-543 0.32 0.53 N/D N/D 0.95 N/D 1.28 0.86 3.47 1.48 0.82 1.03 0.58 2.51 0.58 1.05 0.39 0.39 4.69 N/D N/D N/D

miR-652 N/D N/D N/D N/D N/D N/D N/D N/D N/D N/D N/D N/D N/D N/D N/D 0.23 0.39 N/D N/D N/D N/D N/D

miR-655 N/D N/D N/D N/D N/D N/D N/D N/D 0.23 N/D N/D N/D N/D N/D N/D 0.12 N/D N/D N/D N/D N/D N/D

miR-744 4.85 5.26 6.49 N/D N/D N/D N/D N/D N/D N/D N/D N/D N/D N/D N/D 0.12 N/D N/D 1.92 0.29 0.42 N/D

miR-1307 2.08 2.58 N/D N/D N/D N/D N/D N/D N/D N/D N/D N/D N/D N/D N/D N/D N/D N/D N/D N/D N/D N/D

PN1 7.78 7.75 7.91 7.63 N/D N/D N/D N/D 3.47 N/D N/D N/D N/D 2.97 4.61 N/D N/D N/D 7.89 2.87 4.22 N/D

PN2 4.48 6.51 1.62 1.14 N/D N/D N/D N/D N/D N/D N/D N/D N/D 2.74 3.17 N/D N/D N/D 0.43 0.72 1.06 N/D

PN3 1.44 N/D 2.03 N/D N/D N/D N/D N/D N/D 0.12 N/D N/D N/D N/D N/D 0.23 N/D N/D 1.92 N/D N/D N/D

PN4 1.86 1.43 N/D N/D N/D N/D N/D N/D N/D N/D N/D N/D N/D N/D N/D N/D N/D N/D N/D N/D N/D N/D

PN5 1.81 N/D 2.84 N/D 0.47 N/D N/D 0.43 N/D N/D N/D N/D N/D N/D N/D 0.35 0.39 N/D N/D 1.00 1.48 N/D

PN6 1.81 1.96 2.43 N/D N/D N/D N/D N/D N/D N/D N/D N/D N/D N/D N/D N/D N/D N/D N/D N/D N/D N/D

PN7 1.49 2.05 0.61 N/D N/D N/D N/D N/D N/D N/D N/D N/D N/D N/D N/D N/D N/D N/D N/D N/D N/D N/D

PN8 1.44 N/D 0.41 3.43 N/D N/D N/D N/D N/D N/D N/D N/D N/D N/D N/D N/D N/D N/D N/D N/D N/D N/D

PN9 1.28 N/D 4.87 N/D N/D N/D N/D N/D N/D N/D N/D N/D N/D N/D N/D N/D N/D N/D N/D N/D N/D N/D

PN10 1.17 1.07 1.22 1.53 N/D N/D N/D N/D 0.23 N/D N/D N/D N/D N/D N/D N/D N/D N/D 0.64 N/D N/D N/D

PN11 1.07 N/D 3.65 N/D N/D N/D N/D N/D N/D N/D N/D N/D N/D N/D N/D N/D N/D N/D N/D N/D N/D N/D

PN12 1.07 N/D 2.23 N/D N/D N/D N/D N/D N/D N/D N/D N/D N/D N/D N/D N/D N/D N/D N/D N/D N/D N/D

### Transcriptome profiles were created for specific developmental stages throughout skeletal muscle development in swine. Satellite cells from swine skeletal muscle were evaluated at passage 4, 5, and 6. Data is presented for each individual library and as the average across all satellite cell libraries (Satellite cell average). Transcriptome profiles were also created during fetal development at day 60 (d60), 90 (d90), and 105 (d105) of gestation. Skeletal muscle samples evaluated included biceps femoris (BF) and longissimus dorsi (LD). Muscle samples were also collected from male (M) and female (F) fetuses. Two libraries (F BF d90 1^st^ run, F BF d90 2^nd^ run) were created from the d90 sample of the F BF. Data are presented for each individual library and the average of the two libraries (F BF d90 average). Additionally, libraries were created from skeletal muscle of a day-old neonate swine and an adult female. Two libraries (Adult F BF 1^st^ run, Adult F BF 2^nd^ run) were created for the adult female. Data are presented for each individual library and the average of the two libraries (Adult F BF average). The abundance levels are defined as number identified per 1000 putative miR sequenced for each individual library. The instances of miR168a are known to be a cross-contaminant and do not count toward the total. Included are all miR observed at least 10 times and having a minimum abundance level of 1 per thousand in at least one of the fifteen libraries evaluated. MiR not detected are identified as N/D.
