# Supplementary material for: MicroRNA transcriptome profiles during swine skeletal muscle development
Source: BMC Genomics. 2009 Feb 10;10:77. doi: 10.1186/1471-2164-10-77 (PMC2646747; doi:10.1186/1471-2164-10-77)
Supplement: Additional file 2 — miR-206 sequence variation. The data provided represent the variation of miR-206 sequences identified in the current study. [file 1471-2164-10-77-S2.docx]

**Additional file 2.** MiR-206 sequence variation

| **Sequence** | **Quantity observed** |
| --- | --- |
| UGGAAUGUAAGGAAGUGUGUGA | 2286 |
| UGGAAUGUAAGGAAGUGUGUGAA | 79 |
| UGGAAUGUAAGGAAGUGUGUGU | 78 |
| UGGAAUGUAAGGAAGUGUGU | 41 |
| UGGAAUGUAAGGAAGUGUGUGAU | 34 |
| UGGAAUGUAAGGAAGUGUGUGA | 15 |
| UGGAAUGUAAGGAAGUGUGUG | 13 |
| UGGAAUGUAAGGAAGUGUGU | 9 |
| UGGAAUGUAAGGAAGUGUGUGG | 8 |
| CGGAAUGUAAGGAAGUGUGUGA | 7 |
| GGGAAUGUAAGGAAGUGUGUGA | 5 |
| UGGAAUGUAAGGAAGUGUGUCA | 5 |
| UGGAAUGUAAGGAAGUGUGCGA | 4 |
| UGGAAUGUAAGGAAGUGUGUGUU | 4 |
| UGAAAUGUAAGGAAGUGUGUGA | 3 |
| UGCAAUGUAAGGAAGUGUGUGA | 3 |
| UGGAAUGUAAGGAAGUGCGUGA | 3 |
| UGGAAUGUAAGGAAGUGUGUAA | 3 |
| UGGAAUGUAAGGAAGUGUGUGAAA | 3 |
| UGGAAUGUAAGGAAGUGUGUGC | 3 |
| UUGAAUGUAAGGAAGUGUGUGA | 3 |
| GGAAUGUAAGGAAGUGUGUGA | 2 |
| UGGAAUGUAAGGAAGUGU | 2 |
| UGGAAUGUAAGGAAGUGUAUGA | 2 |
| UGGAAUGUAAGGAAGUGUG | 2 |
| UGGAAUGUAAGGAAGUGUGUAU | 2 |
| UGGAAUGUAAGGAAGUGUGUGAAU | 2 |
| UGUAAUGUAAGGAAGUGUGUGA | 2 |
| AUGGAAUGUAAGGAAGUGUGUGA | 1 |
| AUGGAAUGUAAGGAAGUGUGUGAU | 1 |
| CGGAAUGUAAGGAAGUGUGUGAA | 1 |
| GGGAAUGUAAGGAAGUGUGUGAA | 1 |
| UAGAAUGUAAGGAAGUGUGUGA | 1 |
| UAGAAUGUAAGGAAGUGUGUGAAUGC | 1 |
| UCGAAUGUAAGGAAGUGUGUGA | 1 |
| UGAAAUGUAAGGAAGUGUGUG | 1 |
| UGAAAUGUAAGGAAGUGUGUGU | 1 |
| UGGAAUGUAAGGAAGUGCGUGAA | 1 |
| UGGAAUGUAAGGAAGUGUGGA | 1 |
| UGGAAUGUAAGGAAGUGUGGGA | 1 |
| UGGAAUGUAAGGAAGUGUGUG | 1 |
| UGGAAUGUAAGGAAGUGUGUGAA | 1 |
| UGGAAUGUAAGGAAGUGUGUGAAUG | 1 |
| UGGAAUGUAAGGAAGUGUGUGAG | 1 |
| UGGAAUGUAAGGAAGUGUGUGAU | 1 |
| UGGAAUGUAAGGAAGUGUGUGAUU | 1 |
| UGGAAUGUAAGGAAGUGUGUGU | 1 |
| UGGAAUGUAAGGAAGUGUGUUA | 1 |
| UGGAAUGUAAGGAAGUGUUUGA | 1 |

Mir-206 differed in length or sequence at the 5’ and 3’ ends. MiR-206 sequences of this type were clustered into a single miR sequence identified as the predominant sequence.
