# Supplementary material for: MicroRNA transcriptome profiles during swine skeletal muscle development
Source: BMC Genomics. 2009 Feb 10;10:77. doi: 10.1186/1471-2164-10-77 (PMC2646747; doi:10.1186/1471-2164-10-77)
Supplement: Additional file 3 — Predicted targets of up-regulated miR. The data provided represent the predicted gene targets for the up-regulated miR. [file 1471-2164-10-77-S3.docx]

**Additional file 3.** Predicted targets of up-regulated miR

| miRNA | Hit score | Gene symbol | Gene name | Gene Ontology Function |
| --- | --- | --- | --- | --- |
| miR-206 | 306 | MEF2A | MADS box transcription enhancer factor 2 | Transcription factor activity |
| miR-206 | 160 | CNN3 | Calponin 3 | Actin binding |
| miR-206 | 156 | DMPK | Dystrophia myotonica-protein kinase | ATP binding |
| miR-338 | 161 | GJA1 | Gap junction protein, alpha 1 | Signal transducer activity |
| miR-338 | 150 | SMTN | Smoothelin | Actin binding |

MiR targets were predicted by miRNA viewer for miR-206 and miR-338. For the purposes of this study, the predicted target genes were filtered to include those with muscle listed in the gene ontology and a hit score greater or equal to150. A total of 5 genes were selected based on these criteria
