# Supplementary material for: MicroRNA transcriptome profiles during swine skeletal muscle development
Source: BMC Genomics. 2009 Feb 10;10:77. doi: 10.1186/1471-2164-10-77 (PMC2646747; doi:10.1186/1471-2164-10-77)
Supplement: Additional file 4 — Predicted targets of up-regulated miR. The data provided represent the predicted gene targets for the down-regulated miR. [file 1471-2164-10-77-S4.docx]

**Additional file 4.** Predicted targets of down-regulated miR

| **miRNA** | **Hit score** | **Gene symbol** | **Gene Name** | **Gene Ontology Function** |
| --- | --- | --- | --- | --- |
| miR-15a | 312 | MYH7B | Myosin, heavy chain 7B | ATP binding |
| miR-15a | 294 | MBNL1 | Muscleblind-like (Drosophila) | Double-stranded RNA binding |
| miR-15a | 166 | TAGLN2 | Transgelin 2 | Actin binding |
| miR-15a | 156 | GNAO1 | Guanine nucleotide binding protein (G protein) | GTP binding |
| miR-15a | 152 | LMOD1 | Leiomodin 1 (smooth muscle) | Tropomyosin binding |
| miR-15a | 151 | CNN1 | Calponin 1 | Actin binding |
| miR-15b | 166 | NEBL | Nebulette | Structural constituent of muscle |
| miR-15b | 164 | QKI | Quaking homolog | RNA binding |
| miR-16 | 156 | NEBL | Nebulette | Structural constituent of muscle |
| miR-27a | 152 | ACTA2 | Actin, alpha 2 | ATP binding |
| miR-27a | 151 | EZH2 | Enhancer of zeste homolog 2 (Drosophila) | DNA binding |
| miR-27b | 163 | KCNA1 | Potassium voltage-gated channel | Potassium channel activity |
| miR-27b | 158 | MYH7B | Myosin, heavy chain 7B | ATP binding |
| miR-27b | 155 | QKI | Quaking homolog | RNA binding |
| miR-27b | 154 | HCN4 | Hyperpolarization activated cyclic nucleotide-gated potassium channel 4 | cAMP binding |
| miR-29a | 162 | GDF8 | Growth differentiation factor 8 | Growth factor activity |
| miR-29a | 153 | COL5A3 | Collagen, type V, alpha 3 | Extracellular matrix structural constituent |
| miR-29b | 162 | TPM1 | Tropomyosin 1 | Structural constituent of muscle |
| miR-29b | 152 | KCNIP2 | Kv channel interacting protein 2 | Calcium ion binding |
| miR-29b | 150 | NEBL | Nebulette | Structural constituent of muscle |
| miR-29c | 169 | COL5A3 | Collagen, type V, alpha 3 | Extracellular matrix structural constituent |
| miR-29c | 169 | TPM1 | Tropomyosin 1 | Structural constituent of muscle |
| miR-29c | 151 | NEBL | Nebulette | Structural constituent of muscle |
| miR-29c | 140 | MYH7B | Myosin, heavy chain 7B | ATP binding |
| miR-29c | 140 | MTSS1 | Metastasis suppressor 1 | Actin monomer binding |
| miR-34a | 322 | DAG1 | Dystroglycan 1 | Calcium ion binding |
| miR-34a | 157 | COL6A3 | Collagen, type VI, alpha 3 | Extracellular matrix structural constituent |
| miR-34a | 149 | MTSS1 | Metastasis suppressor 1 | Actin monomer binding |
| miR-34b | 154 | CALD1 | Caldesmon 1 | Actin binding |
| miR-34b | 153 | KRT19 | Keratin 19 | Structural constituent of muscle |
| miR-34c | 160 | QKI | Quaking homolog | RNA binding |
| miR-34c | 155 | KRT19 | Keratin 19 | Structural constituent of muscle |
| miR-34c | 150 | ATP2A2 | ATPase, Ca++ transporting, cardiac muscle, slow twitch 2 | ATP binding |
| miR-106a | 315 | MEF2C | MADS box transcription enhancer factor 2, polypeptide C (myocyte enhancer factor 2C) | Transcription factor activity |
| miR-106a | 172 | SGCE | Sarcoglycan, epsilon | Calcium ion binding |
| miR-106a | 157 | GATA6 | GATA binding protein 6 | Transcription factor activity |
| miR-106a | 151 | QKI | Quaking homolog | RNA binding |
| miR-106a | 150 | FHL1 | Four and a half LIM domains 1 | Molecular function |
| miR-106b | 297 | QKI | Quaking homolog | RNA binding |

MiR targets for miR-15, miR-16, miR-27, miR-29, miR-34, and miR-106 were predicted by miRNA viewer. For the purposes of this study, the predicted target genes were filtered to include those with muscle listed in the gene ontology and a hit score greater or equal to 140. A total of 25 genes were selected based on these criteria, with many targeted by multiple miR.
